# Characterization of a Natural Accession of *Elymus sibiricus* with *In Situ* Hybridization and Agronomic Evaluation

**DOI:** 10.3390/plants14010075

**Published:** 2024-12-29

**Authors:** Yizhuo Liu, Jiarui Ding, Chunfei Wu, Weiwei Song, Xinyu Zhao, Haibin Zhao, Yunfeng Qu, Hui Jin, Rui Zhang, Mingyao Li, Xinyu Yan, Liangyu Zhu, Yaqi Bao, Dianhao Liu, Xinling Li, Lei Cui, Hongjie Li, Yanming Zhang

**Affiliations:** 1 Key Laboratory of Molecular Cytogenetics and Genetic Breeding of Heilongjiang Province, College of Life Science and Technology, Harbin Normal University, Harbin 150025, China; lyz@stu.hrbnu.edu.cn (Y.L.); 15845412785@163.com (J.D.); 13349455229@163.com (C.W.); 1147853055@stu.hrbnu.edu.cn (W.S.); 815736365@stu.hrbnu.edu.cn (X.Z.); 17603660181@163.com (M.L.); hsdyxy0425@stu.hrbnu.edu.cn (X.Y.); zhuliangyu01@163.com (L.Z.); 2024300929@stu.hrbnu.edu.cn (Y.B.); 15048613726@163.com (D.L.); lixinling2002@126.com (X.L.); 2 Institute of Forage and Grassland Sciences, Heilongjiang Academy of Agricultural Sciences, Harbin 150086, China; hbzhao0617@163.com (H.Z.); jinhuicaas@126.com (H.J.); zr0705@126.com (R.Z.); 3 Institute of Biotechnology, Xianghu Laboratory, Hangzhou 311200, China; quyunfeng@xhlab.ac.cn; 4 College of Agriculture, Shanxi Agricultural University, Taiyuan 030031, China; cuilei_sxaas@126.com

**Keywords:** *Elymus*, smGISH, karyotype, chromosome, cold-hardiness

## Abstract

*Elymus sibiricus*, valued for its perennial nature, broad adaptability, strong cold tolerance, and high economic value in forage production, plays a crucial role in combating grassland degradation, desertification, and salinization. Using morphological and cytogenetic methods, this study evaluated the cold tolerance, post-harvest regeneration capacity, and perennial characteristics of the *E. sibiricus* accession 20HSC-Z9 in the Harbin region of China from 2020 to 2023. This accession exhibited a germination rate of over 90% and a 100% green-up rate, with purple coleoptiles indicating its strong cold tolerance. Over the three growing seasons, 20HSC-Z9 maintained stable green-up and regeneration rates, confirming its perennial nature. Morphologically, 20HSC-Z9 had an average tiller count ranging from 56 to 74, similar to that of the control accession 20HSC-ES, and its plant height was significantly lower than that of 20HSC-IWG. Furthermore, 20HSC-Z9 produced over 100 grains per spike, with a seed setting rate exceeding 90%, and a thousand-grain weight comparable to that of 20HSC-IWG. The grain protein content of 20HSC-Z9 reached a maximum of 21.19%, greater than that of the control accessions (15.6% and 18.5%). Chromosome composition analysis, using sequential multicolor genomic *in situ* hybridization and multicolor fluorescence *in situ* hybridization, confirmed the StStHH genomic constitution of 20HSC-Z9 and revealed translocations between the St and H subgenome chromosomes. These results suggest that 20HSC-Z9 has significant potential as a new perennial forage grass germplasm for cold regions, suitable for further domestication and breeding efforts.

## 1. Introduction

*Elymus* L., a perennial genus within the Triticeae tribe of Poaceae, comprises approximately 150 species globally [1,2]. It is predominantly found in temperate regions of the northern hemisphere, particularly in East Asia and North America, with fewer species in Europe. In China, it is widely distributed across the northeast, northwest, and Qinghai-Tibet Plateau. As an important member of the tribe Triticeae, *Elymus* serves as an important gene pool for disease and stress resistance, adaptability enhancement, and high-quality protein sources, demonstrating significant value for wheat improvement [3,4,5,6]. Additionally, *Elymus* is highly regarded as a superior forage grass due to its perennial nature, broad adaptability, strong cold tolerance, and economic value, making it crucial in combating grassland degradation, desertification, and salinization [7].

The genome of *Elymus* is complex, with chromosome numbers varying from tetraploid to octaploid (2n = 4x, 6x, 8x = 28, 42, 56) [8,9,10,11]. Allopolyploids within the genus, containing subgenomes St, H, W, P, and Y, have been categorized by Löve [1] and Dewey [3]. The St genome, originating from the diploid *Pseudoroegneria strigosa* (Schult.) Á. Löve (2n = 2x = 14, StSt), is a fundamental subgenomic component across all *Elymus* species [12]. The H genome is derived from the diploid *Hordeum bogdanii* Wilensky (2n = 2x = 14, HH), the W genome originates from *Australopyrum coinitum* (Wallr.) St.-Yih & H. Ohki (2n = 2x = 14, WW), and the P genome from *Agropyron cristatum* (L.) Gaertn. cv. Fairway (2n = 2x = 14, PP) [13,14,15]. The Y genome’s donor remains unclear, with the possibility that St and Y share a common ancestor [16], or that the Y genome could have evolved from the St genome [12].

*Elymus sibiricus* L. (2n = 4x = 28, HHStSt), an allotetraploid species, commonly known as Siberian wildrye, is native to Europe and northern Asia and is extensively distributed across Eastern Europe, Asia, and North America [17,18]. Due to its robust environmental adaptability and high feed yield, *E. sibiricus* is widely utilized as a forage crop in northwestern Canada and southern Alaska [19]. In China, it is recognized for its strong cold tolerance, vigorous tillering, abundant foliage, excellent palatability, and high protein content, making it an ideal candidate for breeding high-quality forage varieties and for grassland restoration [20]. Using principal component analysis, Ntakirutimana et al. [21] revealed that the final impact of awn length on the seed yield of *E. sibiricus* depends on the balance of its positive and negative effects on traits determining seed yield.

Over the last decade, Dou et al. [22] and Xie et al. [23] have established the reference karyotype map of *E. sibiricus* using fluorescent *in situ* hybridization (FISH) with specific probes, including (AAG)10, pAs1, 5SrDNA, 45SrDNA, and pSc119.2, which have been identified as probes specific to *E. sibiricus* chromosomes. It was observed that chromosomes of the H subgenome in *E. sibiricus* exhibited more variability than those of the St subgenome. Additionally, chromosomal variation was found to correlate closely with geographical distribution changes [23]. In a related study, using wheat cDNA sequences as FISH probes, 32 chromosomal variants were identified in *E. sibiricus* sourced from four different locations, with 19 and 13 variants located on H- and St-subgenome chromosomes, respectively. The highest frequency variants occurred on chromosomes 1H, 2H, 4H, and 2St [24]. Despite the identification of 20 dehydration responsive element (CBF)-genes in barley (*H. vulgare* L.), research on the cold tolerance mechanism of *E. sibiricus* is still incomplete due to the lack of reference genome information [25].

This research identified a natural variation of *E. sibiricus*, designated 20HSC-Z9, in the forage germplasm field, which was investigated for three consecutive years. As a new germplasm, 20HSC-Z9 exhibits the same cold tolerance as the hexaploid *Thinopyrum intermedium* (Host) Barkworth & D. R. Dewey (2n = 6x = StStJ^r^J^r^J^vs^J^vs^) and superior yield composition in Harbin, Heilongjiang province, China. This study aimed to analyze the agronomic characteristics and chromosome composition of 20HSC-Z9 using cold tests, morphological observations, sequential multicolor genomic *in situ* hybridization (smGISH), multicolor fluorescence *in situ* hybridization (mcFISH), and karyotyping. The objective was to deepen the understanding of the genetic variation in *E. sibiricus* and to provide new germplasm resources for breeding perennial grasses adapted to cold regions.

## 2. Results

### 2.1. Cold-Hardiness and Post-Harvest Regrowth of Line 20HSC-Z9

**Figure 1:**
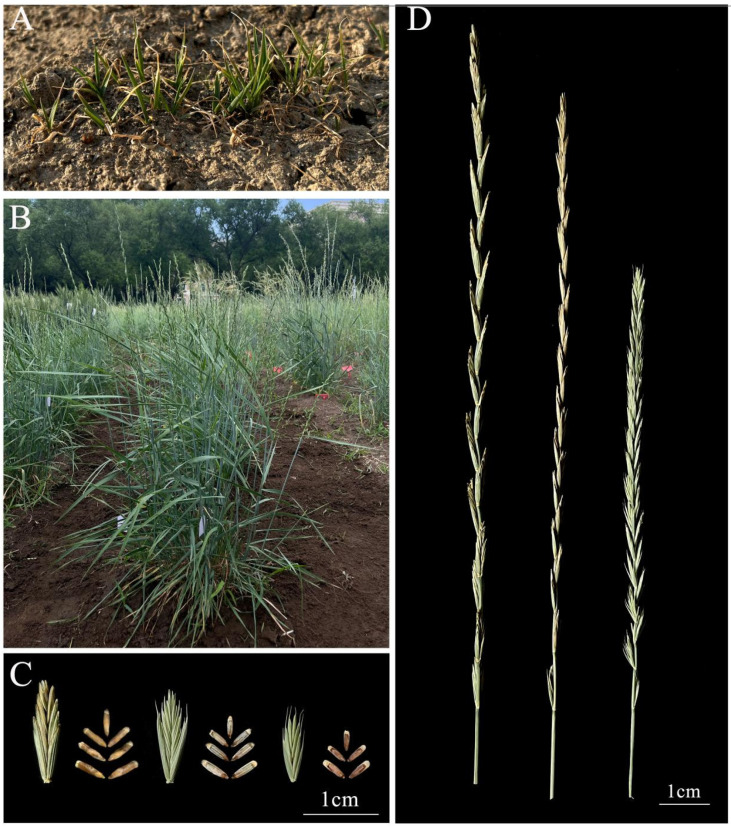
Observation of plant growth and morphological characteristics of 20HSC-Z9. (**A**) Spring regrowth of 20HSC-Z9 (photo taken in March 2023). (**B**) Maturity stage of 20HSC-Z9 (photo taken in July 2023). (**C**) Spikelet yield comparison: *Th. intermedium* accession 20HSC-IWG (left), 20HSC-Z9 (middle), and 20HSC-ES (right) (photo taken in October 2023). (**D**) Spike comparison: *Th. intermedium* accession 20HSC-IWG (left), 20HSC-Z9 (middle), and 20HSC-ES (right). Scale bars = 1 cm.

Line 20HSC-Z9 exhibited cold tolerance and perennial characteristics in Harbin from 2020 to 2023. Sown in the experimental field plots in September 2020, the seedlings emerged with purple coleoptiles at a 90% rate. Plant growth slowed in November, entering dormancy in December under snow cover. Throughout the winter, from November to the end of March, the stems and leaves around the crowns remained green, with new rhizomes appearing in the soil. By early April, the line had returned to a vegetative state with a 100% green-up rate for a new growth cycle (Figure 1A), flowered for a week starting in mid-June, and matured by the end of July (Figure 1B).

Post-harvest regeneration was observed after cutting the aboveground parts in early August. From September to October, new stems and leaves emerged from the crowns. The line maintained a stable rate of green and regeneration after cutting in the subsequent two growth years, confirming its perennial nature. However, underground rhizomes similar to those of the *Th. intermedium* accession 20HSC-IWG have not been observed. Line 20HSC-Z9 is currently in its fourth growth cycle in the field.

### 2.2. Performances of Morphological and Yield-Related Traits

**Figure 2:**
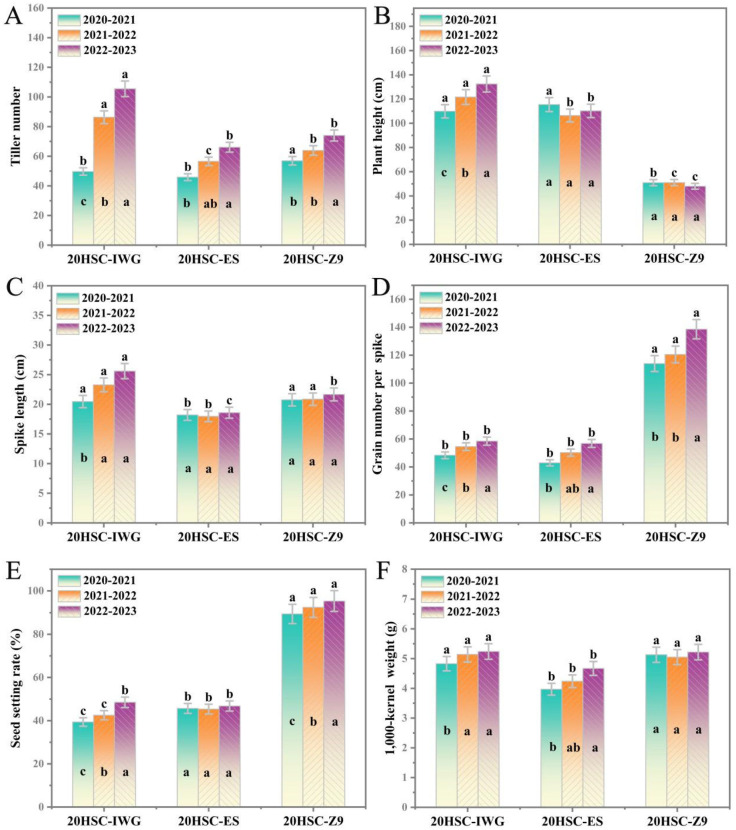
Assessment of agronomic traits, including tiller number (**A**), plant height (**B**), spike length (**C**), grain number per spike (**D**), seed setting rate (**E**), and 1000-kernel weight (**F**) for line 20HSC-Z9 and control lines 20HSC-IWG and 20HSC-ES over three growing seasons (2020–2021, 2021–2022, and 2022–2023) in Harbin. Different letters above the bars indicate significant differences among lines for each trait per season using Fisher’s least significant difference (LSD) test at *p* < 0.05. Different letters inside the bars indicate significant differences among growing seasons for the tested variables of each line at *p* < 0.05.

Over three consecutive growing seasons, line 20HSC-Z9 showed short plant stature, dense spikelets, and excellent yield performance. The tiller number was consistent with the control line 20HSC-ES, averaging 56 to 74 tillers (Figure 2A, Table 1).

The average plant height of line 20HSC-Z9 ranged from 43.2 cm to 48.7 cm, which was significantly lower than that of 20HSC-IWG (131.8 cm) (Figure 2B, Table 1). The average number of spikelets per spike ranged from 23 to 28, with three to five florets per spikelet (Figure 1C, Table 1). The average spike length ranged from 13.2 cm to 21.6 cm (Figure 1D and Figure 2C, Table 1).

Line 20HSC-Z9 had a higher number of grains per spike, seed setting rate, and 1000-kernel weight than the control lines 20HSC-ES and 20HSC-IWG. The number of grains per spike remained above 100, peaking at 138, which was higher than that in 20HSC-ES (56) and 20HSC-IWG (58) (Figure 2D, Table 1). The average seed set percentage for 20HSC-Z9 was approximately 90%, with a peak of 95.4% (Figure 2E, Table 1). The 1000-kernel weight in 2023 exceeded 5 g, matching that of 20HSC-IWG (Figure 2F, Table 1). The average grain length and width of 20HSC-Z9 were 3.1–3.8 mm and 1.3–1.5 mm), respectively, similar to those of 20HSC-IWG (4.1 mm and 1.4 mm), and higher than those of 20HSC-ES (2.7 mm and 1.1 mm) (Figure 1C, Table 1). Additionally, the protein content of 20HSC-Z9 exceeded 20%, higher than that of 20HSC-ES (15.62%) and of 20HSC-IWG (18.51%) (Table 1).

### 2.3. Identification of Chromosome Composition in Line 20HSC-Z9 Using smGISH and mcFISH

**Figure 3:**
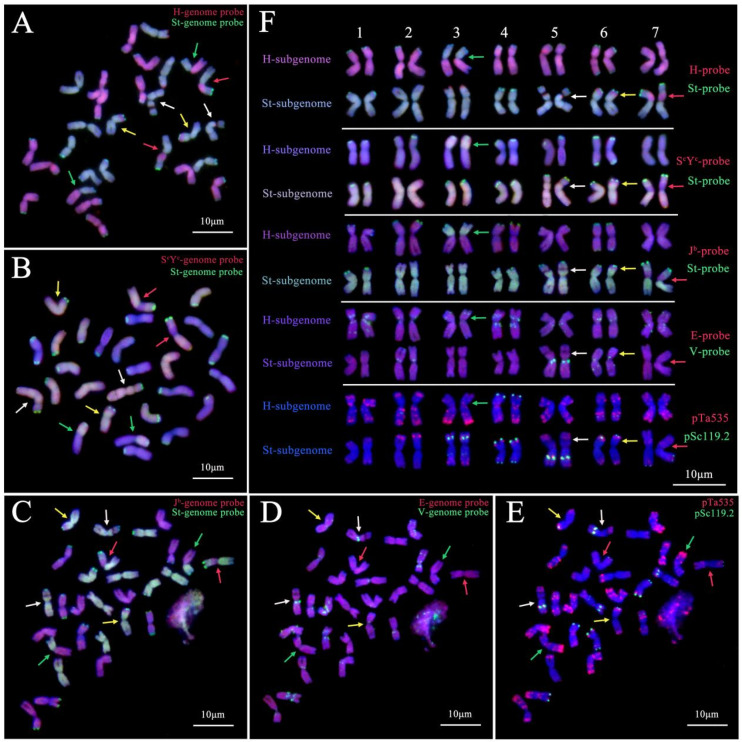
Karyotype analysis of 20HSC-Z9 (StStHH, 2n = 4x = 28) using smGISH and mcFISH. (**A**) Probe combination of *H. bogdanii* (H genome, red) and *Ps. strigosa* (St genome, green). (**B**) Probe combination of *R. ciliaris* (S^c^Y^c^ genome, red) and *Ps. strigosa* (St genome, green). (**C**) Probe combination of *Th. bessarabicum* (J^b^ genome, red) and *Ps. strigosa* (St genome, green). (**D**) Probe combination of *Th. elongatum* (E genome, red) and *D. villosum* (V genome, green). (**E**) Probe combination of *pSc*119.2 (green) and *pTa*535 (red). Patterns in (**C**–**E**) are from the same root tip cells. (**F**) smGISH analysis of 20HSC-Z9 under various probe combinations. Arrows indicate different chromosomal variations: green for whole arm translocations of St-H chromosomes, yellow for small segment translocations of the H-St chromosomes at the short arm, red for large segment translocations of H-St chromosomes at the short arm, and white for chromosomes with mutation loci. Chromosome separation is indicated by white vertical lines. Chromosomes are counterstained with DAPI (blue). Scale bars = 10 μm.

Analysis of line 20HSC-Z9 using genomic DNA from the diploid species *H. bogdanii* (H genome, red) and *Ps. strigose* (St genome, green) as probes revealed that 10 chromosomes of the St subgenome exhibited complete light green fluorescence signals. Two pairs of the St subgenome chromosomes showed small fragment translocations with the H subgenome chromosomes, primarily at the proximal end or near the centromere of the short arm (Figure 3A,F).

One pair of St subgenome chromosomes displayed red hybridization signals in the middle of the short arm using the H genome probe. Four pairs of St subgenome chromosomes had bright hybridization signals from the St genome probe at the chromosome ends. Among the 14 chromosomes of the H subgenome chromosomes with dark pink fluorescence signals, one pair showed a whole-arm translocation with St subgenome chromosomes. Twelve chromosomes had a light green fluorescence signal at the ends, indicating the presence of the St subgenome chromosomes (Figure 3A,F). Thus, the chromosome composition of 20HSC-Z9 was determined to be StStHH, with three pairs of chromosomes exhibiting fragment translocations between St and H subgenome chromosomes (Figure 3A,F).

To confirm the genetic composition, line 20HSC-Z9 was analyzed using genomic DNA probes from the diploid species *Th. bessarabicum* (J^b^ genome, red), *Th. elongatum* (E genome, red), and *D. villosum* (V genome, green), as well as the tetraploid species *R. ciliaris* (S^c^Y^c^ genome, red). The results of smGISH distinguished St and H subgenome chromosomes, with varied mixed probe hybridization signals. The S^c^Y^c^ genome probe labeled St and H subgenome chromosomes of 20HSC-Z9 with pink and lilac fluorescence signals, respectively (Figure 3B,F). The red-colored fluorescence signals stained by probes of the *Th. bessarabicum* (J^b^) and *Th. elongatum* (E) genomes stained H subgenome chromosomes in 20HCS-Z9, but without a specific pattern (Figure 3C,D,F). The V genome probe’s green fluorescence signal was distributed as dots along the long or short arms and near the centromeres of the StH subgenome chromosomes, with a greater distribution on H subgenome chromosomes (Figure 3D,F).

FISH analysis with oligonucleotides *pSc119.2* (green) and *pTa535* (red) as probes showed that *pSc119.2* signals were primarily localized at the terminal, proximal, and medial regions of five pairs of St subgenome chromosomes and the terminal regions of one pair of H subgenome chromosomes. The *pTa535* probe exhibited hybridization signals on all St and H subgenome chromosomes, with a predominance in terminal regions and more abundant signals on H subgenome chromosomes (Figure 3E,F).

Combined smGISH and mcFISH analysis confirmed that the 20HSC-Z9 chromosome set carried both St and H subgenomes with 14 complete chromosomes. Chromosome variation was observed between the two subgenomes, with mutated chromosomes potentially originating from specific regions: the proximal end of the short arm of chromosome 3 in the St subgenome, the vicinity of the centromere on the short arm of chromosome 7 in the St subgenome, and the short arm of chromosome 7 in the H subgenome (Figure 3A,F). The application of genome probes for diploid species *Th. bessarabicum* (J^b^ genome, red), *Th. elongatum* (E genome, red), and *D. villosum* (V genome, green) revealed homologous relationships between the H genome and the J^b^ and E genomes, and a homologous sequence of the V genome on the St and H genomes.

### 2.4. Homologous Group Analysis of Line 20HSC-Z9 with ISH Karyotype

**Figure 4:**
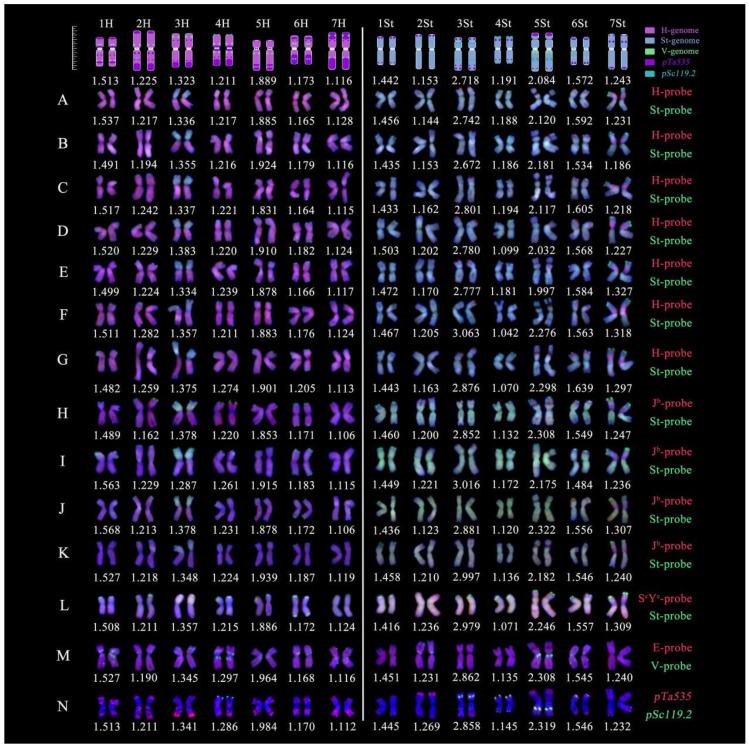
Karyotype analysis of 20HSC-Z9 (StStHH, 2n = 4x = 28) with reference pattern plots for *H. bogdanii* (H genome) and *Ps. strigosa* (St genome) on the top. The figure displays the chromosome signals from five genomic probes, H (red), J^b^ (red), E (red), S^c^Y^c^ (red), St (green), and V (green), as well as two oligonucleotide probes *pTa*535 (red) and *pSc*119.2 (green), with specific colors indicating their presence on chromosomes. (A–G) *H. bogdanii* (H genome, red) and *Ps. strigosa* (St genome, green). (H–K) *Th. bessarabicum* (J^b^ genome, red) and *Ps. strigosa* (St genome, green). (L) *R. ciliaris* (S^c^Y^c^ genome, red) and *Ps. strigosa* (St genome, green). (M) *Th. elongatum* (E genome, red) and *D. villosum* (V genome, green). (N) *pSc*119.2 (green) and *pTa*535 (red). (A–N) are derived from the comparison and arrangement of chromosome arm ratios with reference values. Scale bars = 10 μm, with each unit representing 0.5 μm.

Using reference genomic data from barley (H subgenome) and intermediate wheatgrass (St subgenome), we delineated H and St subgenome karyotypes as reference standards. The arm ratios of the reference karyotype for the H subgenome chromosomes varied from 1.513 (1H) to 1.116 (7H), whereas the ratios for the St subgenome chromosomes varied from 1.442 (1St) to 1.243 (7St) (Figure 4).

Comparing the chromosome arm ratios of 14 cells from 20HSC-Z9 with the reference values, we found that aside from chromosome 5H being a submetacentric (sm), the other H subgenome chromosomes were metacentric (m), with arm ratio variations ranging from 1.482 to 1.568 (1H), 1.162 to 1.282 (2H), 1.287 to 1.383 (3H), 1.211 to 1.297 (4H), 1.831 to 1.984 (5H), 1.164 to 1.205 (6H), and 1.106 to 1.128 (7H), respectively. Chromosomes 3St and 5St, which bear satellites at the end of the short arms, were identified as submetacentric chromosomes (sm), with arm ratio variations ranging from 2.672 to 3.063 (3St) and 1.997 to 2.322 (5St), respectively. The remaining five St subgenome chromosomes were metacentric (m), with arm ratio variations ranging from 1.416 to 1.503 (1St), 1.123 to 1.269 (2St), 1.042 to 1.194 (4St), 1.484 to 1.639 (6St), and 1.186 to 1.327 (7St), respectively (Figure 4). Karyotype analysis of the 392 chromosomes from the 14 cells of 20HSC-Z9 revealed that the third homoeologous group chromosomes of the H subgenome underwent whole arm translocations with chromosomes, and the sixth and seventh homoeologous group chromosomes of the St subgenome experienced small segmental introgressions of the H subgenome chromosomes (Figure 4A–N).

The V genome probe exhibited punctate signals in the centromeric regions of seven pairs of H subgenome chromosomes and chromosomes 2St, 5St, and 6St (Figure 4M). The green signals from *pSc119.2* were primarily distributed in the proximal regions of the short and long arms of chromosomes 4H, 3St, 4St, 5St, 6St, and 7St, while the red signals from *pTa535* were carried by both H and St subgenome chromosomes. The red signals on the H subgenome chromosomes were more abundant and distributed along the short and long arms, as well as the centromeric regions. These signals from the three probes serve as specific markers for the precise differentiation of homoeologous group chromosomes for H and St subgenomes (Figure 4).

## 3. Discussion

The demand for forage yield, cold tolerance, and perennial growth traits has increased for the ecological restoration of grasslands. Developing high-yielding forage varieties capable of growing under extreme conditions is crucial for restoring cold-region pasture ecosystems [24,26]. Over the past decades, various breeding methods, including marker-assisted breeding, genomics, and transgenic technologies, have been applied to develop cold-tolerant forage varieties [27], such as perennial ryegrass (*Lolium perenne* L.) [28], alfalfa (*Medicago sativa* L.) cold-tolerate variety Apica (ATF0) and its derived populations (ATF5) [29], and *Trifolium pratense* L. [30]. Several cultivated varieties of *E. sibiricus*, such as Kangba, Chuancao 2, and Yajiang [31], have been developed by Sichuan Ganzi Prefecture Institute of Animal Science, Sichuan Academic of Grassland Science, and Sichuan Agricultural University, Chengdu, China, respectively, through the domestication and breeding of wild species.

Cold tolerance is essential for the sustained growth of perennial crops in cold regions, allowing them to maintain vitality under the cold climatic conditions of winter and transition into the growth phase when temperatures rise, thus completing their life cycle [32]. In this study, the breeding site was in Harbin, northeastern China (126°89′ E, 46°61′ N), which experiences five months of winter with the lowest temperatures dropping usually to −30 °C. Line 20HSC-Z9 demonstrated a high emergence rate when sown in autumn and successfully overwintered in Harbin, showing promising cold tolerance and sustainable growth over three years. Its yield traits were exceptional, with the number of grains per spike consistently above 100 and a setting rate exceeding 80%. The thousand-grain weight of 20HSC-Z9 exceeded 5 g, which was higher than that of *E. sibiricus* varieties, such as Qingmu 1 and Chuancao 2 [33]. The protein content of the grain reached 21.19%, which was significantly higher than that of 20HSC-ES and similar to that of hexaploid intermediate wheatgrass. Therefore, line 20HSC-Z9 is a promising resource for breeding perennial forage grasses in cold regions.

As a tetraploid model species, *E. sibiricus* possesses the St and H subgenomes derived from diploid *Ps. strigosa,* and barley, respectively [3]. The results of this smGISH analysis for line 20HSC-Z9 align with previous results [23,24], indicating a higher propensity for variation in the H subgenome’s first, second, and fourth homoeologous groups. Translocations were also detected between the H and St subgenome chromosomes, involving particularly the third homoeologous group of the H subgenome and the sixth and seventh homoeologous groups of the St subgenome. Variations in *E. sibiricus* and breeding environments may contribute to these chromosomal structural differences. The presence of satellites on the short arms of the third and fifth homoeologous group chromosomes from the St subgenome of 20HSC-Z9 echoes a previous result in *Ps. strigosa* (St), suggesting conserved chromosomal structures across *Elymus* and *Pseudoroegneria* species.

Regarding the evolutionary relationship, the J^b^ and E genomes are implicated in the evolution of the barley H genome [11]. This study found that probes from diploid *Th. bessarabicum* (J^b^) and *Th. elongatum* (E) genomes stained purple signals on the H subgenome chromosomes of 20HSC-Z9, indicating homology among these subgenomes. The *D. villosum* (V) genome probe’s green punctate signals on the St and H subgenome chromosomes suggest V genome homologous sequences are present on both. Analysis with the tetraploid *R. ciliaris* (S^c^S^c^Y^c^Y^c^) and diploid *Ps. strigosa* (St) genome probes revealed identical hybridization signals on the St subgenome chromosomes of 20HSC-Z9, indicating a high degree of homology within the St genome across these species.

Oligonucleotide probes, known for their locus specificity to certain genetic loci, have become an effective tool for analyzing and identifying homologous chromosomes, with widespread application in wheat chromosome analysis and identification [34]. In this study, FISH analysis using wheat-derived Oligo probes revealed distinct hybridization signal distributions between the St and H subgenomes of 20HSC-Z9. The *pSc119.2* probe primarily hybridized with five pairs of St subgenome chromosomes, while the *pTa535* probe hybridized across all chromosomes, with signals predominantly concentrated at the chromosome termini, suggesting higher sequence conservation in these terminal regions [23].

Karyotype analysis is a standard method for distinguishing homoeologous chromosomes [35]. By comparing the arm ratios of 392 chromosomes from 14 cells and integrating reference genomic karyotype data, homoeologous chromosomes were successfully paired within the two subgenomes of line 20HSC-Z9. This clarified the homoeologous groups involved in St and H subgenome translocations and identified specific hybridization signals stained by the V genome probe and the two Oligo probes within the two subgenomes. These specific hybridization signals can serve as fluorescent markers for detecting St and H subgenome chromosomes in the genus *Elymus*. This demonstrates that karyotyping based on smGISH, FISH, and reference genomic chromosomes is an efficient and feasible method for the identification and analysis of homoeologous chromosomes.

## 4. Conclusions

*Elymus sibiricus* line 20HSC-Z9 has demonstrated superior cold tolerance and perennial characteristics in Harbin, with high germination and regeneration rates sustained over three years. This makes it a valuable resource for developing perennial forage grasses in cold regions. This line outperforms control varieties in yield-related traits, including grain number per spike, seed setting rate, and thousand-kernel weight. Its protein content exceeds 20%, which is significantly higher than conventional standards, indicating its potential as a high-quality forage variety.

Chromosomal analysis confirmed the StStHH composition of 20HSC-Z9, with translocations observed between three pairs of chromosomes within the St and H subgenomes. FISH analysis using wheat-derived oligonucleotide probes revealed distinct hybridization signal patterns between the two subgenomes of 20HSC-Z9, providing specific markers for the precise differentiation of homoeologous group chromosomes for H and St subgenomes.

This karyotype analysis, employing smGISH, and FISH, offers an efficient and feasible method for the identification and analysis of homoeologous chromosomes in *E. sibiricus*. This provides a foundation for further genetic research and breeding efforts, contributing to the development of improved forage grasses for cold regions.

## 5. Materials and Methods

### 5.1. Plant Materials

Line 20HSC-Z9, a natural variation of *E. sibiricus*, was selected in the forage germplasm field at the Harbin Normal University (HNU), in Harbin, Heilongjiang province, China. The intermediate wheatgrass *Th. intermedium* accession 20HSC-IWG and the *E. sibiricus* accession 20HSC-ES, both collected from HNU, were used as controls in the field test conducted for morphological analysis.

To prepare probes for detecting specific genome chromosomes or chromosomal segments in 20HSC-Z9, accessions from various species were utilized as DNA sources. These included *H. bogdanii* (HH), *Ps. strigosa* (StSt), *Th. bessarabicum* (JJ = J^b^J^b^), *Th. elongatum* (EE = J^e^J^e^), *Dasypyrum villosum* (VV), *E. sibiricus* (StStHH), and *Roegneria ciliaris* (StStYY/S^c^S^c^Y^c^Y^c^). These species provided the genetic materials necessary to identify H, St, J^b^, E, and V genome chromosomes in line 20HSC-Z9.

### 5.2. Evaluation of Perennial Cold-Hardiness and Agronomic Performances

The agricultural climate of Harbin is characterized as semiarid, with spring drought and semi-humid summers. Between October and April from 2020 to 2023, Harbin experienced sub-zero winter temperatures, averaging from −16.5 °C to −21.26 °C over four consecutive years [32].

Line 20HSC-Z9 was evaluated for cold tolerance and agronomic traits over three consecutive cropping seasons from 2020 to 2023, following the methods described by Liu et al. [32]. A plot (2 m × 4 m) propagation experiment was established in September 2020 at the HNU’s forage experimental field with a row spacing of 30 cm. The plants were observed for regeneration of aboveground and underground rhizomes from August to October each year. From November to early April, the plants were exposed to natural cold environments for cold tolerance testing, and their perennial habits were determined based on three years of cold resistance and regeneration testing.

The agronomic traits of 20HSC-Z9, including plant height (cm), spike length (cm), spikelets number, florets number, tiller number, grain number per spike, seed set percentage, and 1000-kernel weight (g), were statistically investigated from late June to mid-July each year. Five plants in the plot were randomly selected to investigate plant height and spike length. Plant height was measured from the ground level to the top of a spike, and spike length from the base of a rachis to the top of a spike. The protein content in 500 seeds was measured using a DA7200 multifunctional near-infrared spectrometer (Perten, Alpnach, Switzerland), with each sample repeated three times. Standard practices of irrigation (one before the overwinter stage immediately after defoliation in November, and the other after grain and forage harvesting in July), fertilizers (nitrogen, phosphorus, and potassium), and pest control were applied following the local farming system [36].

### 5.3. Chromosome Preparation

Seeds were germinated at 23.5 °C for 24 h on moist filter paper in Petri dishes. They were then incubated at 4 °C for 48 h before returning to 23.5 °C for an additional 27.5 h. Root tips were treated with ice water at 0–4 °C for 24 h, fixed in Carnoy’s fixative (a mixture of anhydrous alcohol: acetic acid = 3:1, *v*/*v*) for 24 h, and squashed in 45% acetic acid. The chromosome preparations were observed under a phase-contrast microscope (BH-2, Olympus, Tokyo, Japan).

### 5.4. Analyses of smGISH and mcFISH

Genomic DNA was extracted from young leaves of *H. bogdanii*, *Ps. strigosa*, *Th. bessarabicum*, *Th. elongatum*, *D. villosum*, and *R. ciliaris* using the cetyltrimethylammonium bromide (CTAB) method to prepare St, J^b^, E, V, and S^c^Y^c^ genome probes [37]. Five seeds from 20HSC-Z9 were randomly selected for smGISH analysis. The genomic DNA of *Ps. strigosa* and *D. villosum* were labeled with fluorescein-12-dUTP (green, BioNick Labeling System; Invitrogen, Waltham, MA, USA). The genomic DNA of *H. bogdanii*, *Th. bessarabicum*, *Th. elongatum*, and *R. ciliaris* was labeled with tetranmethyl-rhodamine-5-dUTP (red, DIG-Nick Translation Mix, Roche Diagnostics, Mannheim, Germany). The smGISH protocol was described previously by Liu et al. [32]. Following GISH analysis, slides were washed with 2× SSC and subjected to multicolor FISH using synthetic oligonucleotide probes, including Oligo-*pSc*119.2 and Oligo-*pTa*535 [34]. Hybridization signals were visualized using an Olympus BX53 epifluorescence microscope (Olympus, Tokyo, Japan), equipped with appropriate filters to detect DAPI [2-(4-Amidinophenyl)-6-indolecarbamidine dihydrochloride] (blue), Alexa Fluor 488 (519 nm), and Alexa Fluor 594 (618 nm). Photographs were captured with a Canon digital camera (Model EOS D2000) and processed using Photoshop 22.0.0 (Adobe, San Jose, CA, USA).

### 5.5. Homoeologous Group Analysis Based on ISH Karyotype

For karyotype analysis, we utilized the reference genome data of H and St as the standard chromosome karyotypes. The H subgenome sequence data were sourced from Ensembl Plants (Ensembl_v1.2, https://ftp.ebi.ac.uk/ensemblgenomes/pub/release-58/plants/fasta/hordeum_vulgare/dna/, accessed on 21 March 2024), while the St subgenome sequence data were from the US Department of Energy Joint Genome Institute (*Thinopyrum intermedium* v3.1 DOE-JGI, http://phytozome.jgi.doe.gov/, accessed on 20 April 2024). Chromosome lengths from the smGISH pattern of line 20HSC-Z9 were measured using ImageJ 1.54i (National Institutes of Health, Bethesda, MD, USA, https://imagej.net, accessed on 20 April 2024), with the arm ratio calculated as the total chromosome length divided by the length of the short arm (excluding the satellite) [38]. The subgenomic chromosome karyotypes were mapped using Photoshop 22.0.0 (Adobe, San Jose, CA, USA). Five probe signals, i.e., *H. bogdanii* (H genome, light pink signal), *Ps. Strigose* (St genome, azure signal), *D. villosum* (V genome, green signal), oligonucleotide probes Oligo-*pSc119.2* (light blue signal), and Oligo-*pTa535* (purple signal), were added to the H and St subgenomes as the specific signals of line 20HSC-Z9 to distinguish each pair of homoeologous group chromosomes.

### 5.6. Statistical Analysis

Agronomic traits were analyzed using analysis of variance (ANOVA), with genotype, year, and genotype-by-year interaction as the main factors. Fisher’s least significant difference (LSD) was applied for multiple comparisons to differentiate significant differences among genotypes and plant ages (i.e., the first-, second=, and third-year plants) based on the means of each parameter. Statistical analyses were conducted using IBM SPSS 19 (SPSS Inc., Chicago, IL, USA). Significant differences in growth years and various traits of different materials were identified using a *t*-test at *p* < 0.05.

## Figures and Tables

**Table 1:** Agronomic traits, grain morphological characteristics, and protein content for 20HSC-Z9, 20HSC-ES, and 20HSC-IWG in 2023.

Lines	Years of Growth	Plant Height (cm)	Spike Length (cm)	Spikelet Number	Tiller Number	Grain Number per Spike	Thousand-Kernel Weigh (g)	Seed Setting Rate (%)	Yield Quadrat Size (m^2^)	Seed Length (mm)	Seed Width (mm)	Protein Content (%)
20HSC-Z9	3	48.8	21.7	26.0	74.0	138.6	5.2	95.4	0.024	3.8	1.5	21.2
20HSC-ES	3	121.4	18.0	18.5	66.0	56.8	4.7	46.8	0.020	2.8	1.3	15.6
20HSC-IWG	3	131.8	25.6	23.1	105.5	58.5	5.3	48.5	0.093	4.1	1.4	18.5

## Data Availability

All data generated or analyzed during this study are included in this published article.
